# Health Evaluation and Referral Assistant: A Randomized Controlled Trial of a Web-Based Screening, Brief Intervention, and Referral to Treatment System to Reduce Risky Alcohol Use Among Emergency Department Patients

**DOI:** 10.2196/jmir.6812

**Published:** 2017-05-01

**Authors:** Brianna L Haskins, Rachel Davis-Martin, Beau Abar, Brigitte M Baumann, Tina Harralson, Edwin D Boudreaux

**Affiliations:** ^1^ University of Massachusetts Medical School Worcester, MA United States; ^2^ University of Rochester Medical Center Rochester, NY United States; ^3^ Cooper Medical School of Rowan University Camden, NJ United States; ^4^ Polaris Health Directions, Inc Wayne, PA United States

**Keywords:** alcohol consumption, intervention study, emergency medicine, referral and consultation

## Abstract

**Background:**

Computer technologies hold promise for implementing alcohol screening, brief intervention, and referral to treatment (SBIRT). Questions concerning the most effective and appropriate SBIRT model remain.

**Objective:**

The aim of this study was to evaluate the impact of a computerized SBIRT system called the Health Evaluation and Referral Assistant (HERA) on risky alcohol use treatment initiation.

**Methods:**

Alcohol users (N=319) presenting to an emergency department (ED) were considered for enrollment. Those enrolled (n=212) were randomly assigned to the HERA, to complete a patient-administered assessment using a tablet computer, or a minimal-treatment control, and were followed for 3 months. Analyses compared alcohol treatment provider contact, treatment initiation, treatment completion, and alcohol use across condition using univariate comparisons, generalized estimating equations (GEEs), and post hoc chi-square analyses.

**Results:**

HERA participants (n=212; control=115; intervention=97) did not differ between conditions on initial contact with an alcohol treatment provider, treatment initiation, treatment completion, or change in risky alcohol use behavior. Subanalyses indicated that HERA participants, who accepted a faxed referral, were more likely to initiate contact with a treatment provider and initiate treatment for risky alcohol use, but were not more likely to continue engaging in treatment, or to complete treatment and change risky alcohol use behavior over the 3-month period following the ED visit.

**Conclusions:**

The HERA promoted initial contact with an alcohol treatment provider and initiation of treatment for those who accepted the faxed referral, but it did not lead to reduced risky alcohol use behavior. Factors which may have limited the HERA’s impact include lack of support for the intervention by clinical staff, the low intensity of the brief and stand-alone design of the intervention, and barriers related to patient follow-through, (eg, a lack of transportation or childcare, fees for services, or schedule conflicts).

**Trial Registration:**

International Standard Randomized Controlled Trial Number (ISRCTN): NCT01153373; https://clinicaltrials.gov/ct2/show/NCT01153373 (Archived by WebCite at http://www.webcitation.org/6pHQEpuIF)

## Introduction

### Background

Between 2006 and 2010, excessive alcohol consumption was responsible for 88,000 deaths and an estimated 2.5 million years of potential life lost each year. Risky alcohol use is among the leading preventable causes of death in the United States [1], but it remains highly prevalent and poorly intervened among the emergency department (ED) population. Prevalence rates of risky alcohol use among ED patients exceed the national average, making EDs an ideal location for innovative alcohol intervention efforts [2-5]. Over 130 million patients visit the ED each year [6], with a large percentage of these patients having unrecognized alcohol-related treatment needs (eg, risky drinking, problem drinking, and alcohol dependence). Patients with untreated needs are more likely to be admitted to the hospital and repeatedly rely on ED services [2,7-9]. Furthermore, disadvantaged populations including minorities, immigrants, people without insurance, and low-income households comprise the underserved populations that currently rely disproportionately on EDs for primary care [10] and suffer from alcohol-related concerns at elevated rates [11-12]. ED-originated alcohol intervention efforts have the potential for considerable impact on public health by promoting change in both high-risk and hard-to-reach populations [4,10,13].

This potential has been acknowledged by the latest health care legislation and numerous health care agencies. The Affordable Care Act includes strong incentives for the integration of behavioral health and medical treatment [2,14]. Numerous studies have demonstrated the effectiveness of screening and brief intervention (SBI) programs as well as screening, brief intervention, and referral to treatment (SBIRT) programs aimed at addressing alcohol use problems and treatment needs among ED patients [2,15-18]. As a result, the US Preventive Services Task Force and the Substance Abuse and Mental Health Services Administration have recommended universal SBIRT for alcohol and other substances in general medical settings, including EDs [19,20]. The Centers for Medicare and Medicaid Services and the American Medical Association have authorized billing codes to reimburse SBIRT services for alcohol, tobacco, and illicit drug use [21], and the Centers for Disease Control and Prevention have called for increased alcohol SBI, including systems-level changes to include integration into the electronic health record system [22].

Despite the support of numerous studies and many health agencies [2,14-23], ED-originated alcohol screening rates remain low, with many hospitals only screening alcohol toxicology reports, which do not assess problem-level severity [2,24,25]. Factors likely contributing to these low rates include lack of specialized behavioral health training, competing demands on time and resources inherent to the ED setting, and a primary objective of acute medical care rather than treatment for chronic conditions [5,13,23]. The use of convenient and brief procedures requiring minimal specialized training and focusing on connecting patients with outpatient resources for continued treatment after their ED visit could maximize successful implementation of SBIRT. Behavioral intervention technology advancement (eg, computerized assessments, personalized feedback reports, faxed referrals, electronic health records), holds promise for facilitating the implementation of SBIRT in EDs and a variety of health care settings [26-33]. Such advancements have also allowed for the development of computer-assisted SBIRT models designed to diminish interruptions in clinical care and mitigate clinician burden without sacrificing effectiveness [13,23].

### Objectives

Questions remain concerning the most effective and appropriate SBIRT model. The objective of this study was to assess an innovative Web-based program’s ability to facilitate alcohol SBIRT. The Health Evaluation and Referral Assistant (HERA) is patient-administered on a tablet computer during the ED visit and is modeled after the face-to-face SBIRT screening approach. This study hypothesized that the HERA would improve initiation of specialized outpatient treatment for risky alcohol use and reduce risky alcohol use among ED patients at 3 months postvisit as compared with a minimal intervention control condition.

## Methods

### Previous Reporting

A complete description of the HERA development and randomized controlled trial (RCT) methods were previously published [26,34]. Although the HERA assesses and refers patients to treatment for multiple substances, only results pertaining to alcohol are reported and discussed in this paper. A previous publication reported results for tobacco use [23], and a subsequent paper will address the results pertaining to illicit drug use. This clinical trial was registered with ClinicalTrials.gov as the Dynamic Assessment and Referral System.

### Health Evaluation and Referral Assistant (HERA)

#### Assessment

The HERA is a self-administered patient assessment completed on a tablet computer during the ED visit. The assessment was designed to require no computer literacy beyond the ability to read at the 8th grade reading level and respond to questions using a numeric keypad or stylus. The HERA used the Alcohol Use Disorders Identification Test (AUDIT) to assess alcohol use behaviors [35]. The version was based on the Cutting Back study [36], which adjusted the responses to the first three items of the AUDIT to reflect US alcohol content standards. This version has ultimately become what is referred to as the USAUDIT and has been adopted by the Centers for Disease Control and Prevention [37].

Readiness to change was assessed with an initial question that asked, “Would you like to change your alcohol use? No; Undecided; Yes, I would like to CUT BACK; Yes, I would like to QUIT COMPLETELY.” If interested in quitting, the participant was asked, “When would you like to quit? Within the next 30 days; Within the next 6 months; More than 6 months from now.” Treatment history was assessed by asking, “Have you ever been in treatment for alcohol use? No; Yes, but I AM NOT CURRENTLY in treatment; Yes, and I AM CURRENTLY in treatment.” Readiness to enter treatment was assessed for those who scored in the risky alcohol use range, were not currently in treatment, and reported interest in changing alcohol use by asking, “You have reported that you are interested in changing your alcohol use. This computer program can help you connect with a counselor or treatment program. Would you like some help with finding a counselor or treatment program? Yes; No.” Withdrawal symptoms were assessed using a checklist of items: “Please check all of the withdrawal symptoms you had in the past 30 days, including today: seizures or convulsions; hallucinations (saw, heard, or felt something that was not there); confusion or disorientation; paranoid thinking; severe depression; severe loss of energy (lethargy); none of the above.” The Patient Health Questionaire-2 (PHQ-2) [38] was used to screen participants for depression, and a complete psychiatric history was documented using a checklist of common psychiatric diagnoses (eg, anxiety, post-traumatic stress disorder, bipolar disorder, schizophrenia, attention deficit hyperactivity disorder, and so on). See Multimedia Appendix 1 for sample assessment screenshots.

#### Report Generator

The assessment data were used to automatically produce two reports at the end of the computerized assessment, which are described in detail in the aforementioned manuscripts [26,34]. The health care provider report provided a summary of the assessment and was given to the patient’s treating physician for review. The patient feedback report was given to the patient and consisted of 3 sections: (1) the Face Sheet, which included an overview and tailored alcohol use treatment referral list; (2) the Patient Assessment Summary, which provided individually tailored feedback pertaining to the patient’s alcohol use; and (3) the Motivation Toolkit, which provided several worksheets based on Motivational Interviewing [39] and the Transtheoretical Model [40]. See Multimedia Appendix 2 for a sample patient feedback report.

#### Referral Generator

The referral generator utilized a library of alcohol use treatment services maintained by Polaris Health Directions, Inc. to create individually tailored referral lists and to send dynamic referrals. Referral lists contained free and fee-for-service treatment options, and dynamic referrals were based on a “best match” facility dependent on patient characteristics, such as the individual’s ZIP code, insurance provider, and preference for telephone or in-person treatment. If accepted by the patient, the dynamic referral was faxed by the HERA to a matched treatment facility, along with a brief assessment summary and the patient’s contact information. The participating services had agreed to contact the patient within 48 h of receiving the referral to complete an initial evaluation and discuss treatment options.

### Procedure

Patients were enrolled from 4 EDs (see Table 1) between 8 am and 7 pm, with shifts occurring every day of the week. Research assistants (RAs) approached all adult patients at their bedside during their ED visit. Patients aged 18 years and older with risky alcohol use were considered. Risky alcohol users were defined as having used alcohol above the AUDIT quantity or frequency guidelines, with or without tobacco use but with no illicit drug use in the past 12 months. This paper focuses only on alcohol users who may have been smokers but did not use illicit drugs. Exclusion criteria were severe illness or distress, cognitive insufficiency, in state custody or restraints, being held involuntarily, and language barriers. Patients who were actively involved in alcohol treatment were eligible for the study, but few agreed to participate. Patients were enrolled regardless of whether they were admitted or discharged. The study components, including baseline and intervention, were completed while patients were in the ED. Participants were randomized to either the intervention or control condition by a random number generator from the Java programming language standard library embedded within the HERA. Immediately after discharge or transfer from the ED, the enrolling RA completed a brief interview with the participant, either in person or by telephone within 48 h (postvisit interview). A trained RA not affiliated with the data collection sites contacted all participants by telephone at 1 and 3 months following the ED visit to assess alcohol treatment initiation and to reassess alcohol use. This study was approved by the Institutional Review Boards for all data collection sites, in accordance with the ethical standards of the Helsinki Declaration of 1975. All participants gave their informed consent and signed a written consent form before inclusion in the study.

**Table 1:** Site characteristics. This table was previously published with the reporting of the tobacco results [23].

Type	Annual volume	Location	Race or ethnicity
Academic, urban	90,733	Worcester, MA^a^	W^b^ 82%, H^c^ 11%, B^d^ 4%
Community, urban	47,364	Worcester, MA	W 74%, H 14%, B 9%
Community, suburban	23,217	Marlboro, MA	W 80%, H 15%, B 3%, U^e^ 2%
Academic, urban	59,482	Camden, NJ^f^	W 35%, H 20%, B 45%

^a^MA: Massachusetts.

^b^W: white, non-Hispanic.

^c^H: Hispanic.

^d^B: black.

^e^U: unknown.

^f^NJ: New Jersey.

### Study Conditions

Intervention and control conditions were treated the same in all aspects of the study procedures; however, the groups differed on the type of referral and availability of reports. Participants in the intervention condition (HERA) (1) were offered a dynamic referral, (2) received the patient feedback report with a tailored referral list, and (3) their treating physician received the health care provider report. Participants assigned to the minimal intervention control condition (control) were given a standardized, printed list of local treatment providers instead of dynamic referrals, and health care provider reports were not made available.

### Blinding

The RA who performed the outcome assessments was partially blinded. Because the HERA is heavily focused on the referral process, and not all patients received the same type of referrals, to avoid confusion, the follow-up questions were tailored to the referral type received at baseline (printed list vs dynamic referral). Despite blinding efforts, the presence of particular questions for the intervention group revealed some information about group assignment. For example, only patients who chose a dynamic referral were asked whether they had been contacted by an alcohol treatment provider.

### Measures

#### Health Evaluation and Referral Assistant (HERA)

The HERA assessment was previously described under *Methods* or *Assessment*.

#### Postvisit Interview

Immediately after patients were discharged or transferred from the ED, the enrolling RA completed a brief interview to establish whether the treating clinicians provided alcohol treatment counseling, education materials, or referrals for alcohol use treatment. Chart review was not used because of unreliability associated with documentation.

#### Follow-Up Assessment

All participants were phoned by an RA and asked if they had initiated contact with an alcohol treatment provider or program (treatment contact); completed an initial assessment (treatment initiation); attended any additional treatment sessions beyond the initial assessment (treatment engagement); and completed treatment (treatment completion). Participation in self-help groups, like Alcoholics Anonymous, was also assessed. Additionally, the RA assessed self-reported current alcohol use using the first three items from the USAUDIT (frequency of drinking, amount on a typical day, frequency consuming four or more drinks on a single occasion). This was used to quantify use and to determine abstinence, which was defined as 0 drinks since the ED visit. Efforts to decrease use were assessed with the following questions: “In the past ‘x’ months, have you tried to reduce your alcohol use? Yes; No. In the past ‘x’ months, have you intentionally gone for more than 24 h without having a drink? Yes; No. In the past ‘x’ months, how many days have you gone without having a drink?”

### Data Analyses

Baseline characteristics (eg, demographics, alcohol use) were compared across intervention conditions using chi-square test of independence and independent samples *t* test to confirm randomization success, and the potential for differential retention rates across conditions was examined using chi-square test of independence. Our primary outcomes (ie, alcohol treatment provider contact, treatment initiation, alcohol use) were then compared across conditions at 1 and 3 months using generalized estimating equation (GEE) models. Post hoc chi-square test of independence was performed following a statistically significant GEE model in order to better isolate the observed differences at each follow-up point. Chi-square analyses were also used to make comparisons across conditions for outcomes collected only at a single follow-up point (eg, ED counseling).

We then performed a series of analyses comparing participants in 3 distinct groups: (1) the control condition, (2) the intervention condition that declined a dynamic referral to providers (tailored list only), and (3) the intervention condition that accepted a dynamic referral (dynamic referral group). Because this categorization allows for preexisting differences across groups (particularly between the tailored list and dynamic referral groups), these models included theoretically relevant covariates that might impact the outcomes of interest (baseline AUDIT scores and readiness to quit). Missing data or attrition at follow-up was addressed using standard intention-to-treat principles whereby the least favorable outcome (eg, no provider contact, no treatment completion) was assigned to missing data points. Specifically, if data were missing at both follow-up points for a case, the least favorable outcome was imputed. If data from the first follow-up indicated a favorable outcome (eg, quit attempt, initiated treatment) and data was missing at the second follow-up, a favorable outcome would be imputed as we were interested in the event occurring by a given time point. If data were missing at the first follow-up and present at the second follow-up, regardless of the outcome at the second follow-up, the least favorable outcome would be imputed at the first follow-up. Given the use of these principles, the frequencies presented in each table represent observed data, whereas the percentages represent intention-to-treat estimates. All analyses were performed using Statistical Package for the Social Science 22 (IBM, 2012), with an a priori alpha level of .05.

## Results

### Preliminary Analysis

Of 319 alcohol users who met eligibility criteria and did not report any drug use, 212 individuals were enrolled (see Multimedia Appendix 3). A greater proportion of eligible females (75.7%, 78/103) enrolled in the study than males (62.0%, 134/216), χ²_1_=5.9 *P*=.02, and enrolled individuals were younger, on average (mean 38.1 years; SD 13.4) than nonenrolled individuals (mean 42.3 years; SD 13.9), *t*_317_=2.63, *P*=.009. There were no differences in percentage of enrolled eligible patients across sites, concomitant tobacco use, and insurance status. Of the 212 participants enrolled, 115 were assigned to the control condition and 97 to the intervention condition. At baseline, there were no differences between the two conditions on demographics (see Multimedia Appendix 4), data collection site (*P*=.06), mental health diagnoses (*P*=.19 to .83), AUDIT scores (*P*=.24), or readiness to change (*P*=.10).

Of the analyzed participants, 196 out of 212 (92.5%) completed the postvisit interview, 157 out of 212 (74.1%) completed the 1-month follow-up, and 157 out of 212 (74.1%) completed the 3-month follow-up (see Multimedia Appendix 3). There were no differences between retained individuals and those lost to follow-up on age (*P*=.99; .17, respectively), baseline AUDIT scores (*P*=.62; .34), mental health diagnoses (*P*=.27 to .81; .09 to .74), or readiness to change (*P*=.58; .21). However, at the 3-month follow-up, there were more control individuals retained (95/115; 83.0%) than experimental (62/97; 64%), χ²_1_=9.6, *P*=.002. There were also more female retained at the 3-month follow-up (64/78; 82%) than male (93/134; 69.4%), χ²_1_=4.1, *P*=.04.

### Comparisons on Outcomes of Interest

#### Specialized Alcohol Use Treatment

There were no differences in initial contact between participants and alcohol use treatment provider across conditions (odds ratio, OR 1.04; 95% CI 0.45-2.40; see Table 2). No differences were observed on treatment initiation (*P*=.53), treatment engagement (*P*=.21), and treatment completion rates either (*P*=.31). Among the participants in the HERA, 14/97 (14%) accepted a dynamic referral.

#### Alcohol Use

Sustained abstinence at both follow-up periods was not statistically different across intervention and control conditions (see Table 2). Quit attempts and efforts to reduce alcohol use were more common among control participants than experimental (OR 0.44, 95% CI 0.26-0.77, *P*=.004; OR 0.66, 95% CI 0.51-0.87, *P*=.01, respectively).

**Table 2:** Comparisons between alcohol intervention and control conditions.^a^

Characteristics			Intervention (n=97), n (%)	Control (n=115), n (%)
**ED^b^clinician (MD^c^** or **RN^d^) counseling^e^**				
		MD or RN asked about alcohol use	62 (64)	80 (69.6)
		MD or RN counseled participant to quit	11 (11)	12 (10.4)
		Received educational materials	2 (2)	4 (3.5)
		Received an alcohol abuse referral	1 (1)	4 (3.7)
**Outpatient alcohol abuse treatment**				
	Contact with alcohol abuse treatment provider			
		GEE^f^ odds ratio 1.04 (95% CI 0.45-2.40), *P*=.94		
		Contact at 1 month	7 (7)	10 (8.7)
		Contact at 3 months	13 (13)	13 (11.3)
	Initiated treatment (evaluated by alcohol abuse treatment provider)			
		GEE odds ratio 0.70 (95% CI 0.23-2.15), *P*=.53		
		Treatment initiation at 1 month	3 (3)	7 (6.1)
		Treatment initiation at 3 months	6 (6)	8 (7.0)
	Treatment engagement at either time		3 (3)	8 (7.0)
	Treatment completion		3 (3)	7 (6.1)
**Alcohol use behavior**				
	Used alcohol (since ED visit)			
		GEE odds ratio 0.80 (95% CI 0.30-2.14), *P*=.66		
		Abstinent for 1st month (since visit)	8 (8)	12 (10.4)
		Abstinent for 3 months (since visit)	3 (3)	4 (3.5)
		At least one quit attempt at 1 month	17 (18)	37 (32.2)
		At least one quit attempt at 3 months	30 (30)	58 (50.4)
		Attempted to reduce use at 1 month	25 (26)	45 (39.1)
		Attempted to reduce use at 3 months	33 (34)	57 (49.6)

^a^All percentages and analyses use the intention-to-treat principle of worst outcome for missing values.

^b^ED: emergency department.

^c^MD: doctor of medicine.

^d^RN: registered nurse.

^e^ED clinician behavior assessment included behaviors over and above the materials provided as part of the research study. All patients in both groups had alcohol assessed as part of the study and received a referral list. The control group received a preprinted list, whereas the intervention group received a personally tailored list, as well as a dynamic referral if desired.

^f^GEE: generalized estimating equation.

#### Physician Behavior

Clinician counseling, provision of educational materials, and provision of referrals, beyond those provided as part of the study protocol, were not statistically different across intervention and control conditions (see Table 2).

#### Exploring the Effect of Dynamic Referrals

Supplemental GEE analyses demonstrated large differences across groups on treatment contact. Using dummy codes (control condition as the reference), results indicated that experimental participants who accepted a dynamic referral contacted a provider at a much greater rate than control individuals (OR 7.14, 95% CI 2.33-20.41, *P*<.001; see Table3). Effects on treatment initiation were also significant, with higher rates of initiation among experimental participants who accepted a dynamic referral and control participants (OR 3.92, 95% CI 1.01-15.15, *P*=.05). There were no differences in treatment initiation between experimental individuals who did not accept a dynamic referral and control individuals (OR 0.26, 95% CI 0.05-1.36, *P*=.11). The difference in contact with providers between experimental participants who accepted a dynamic referral and control participants remained significant (*P*=.001) when accounting for baseline readiness to change and baseline AUDIT scores in a post hoc GEE model. The effect of a dynamic referral on treatment initiation was no longer significant in a similar model.

There was a marginally significant effect of group membership on engagement in alcohol treatment, χ²_2_=5.8, *P*=.06 (see Table 3). Although engagement was quite infrequent across all groups, the rate of engagement for those accepting a dynamic referral was more than double the rate observed in the control condition (2/14, 14% vs 8/115, 7.0%; see Table 3). This effect was no longer significant when baseline readiness to change and AUDIT scores were included as covariates (OR control vs tailored list only=0.23, 95% CI 0.02-2.13, *P*=.20; OR control vs dynamic referral=2.14, 95% CI 0.26-17.54, *P*=.48). There were no effects of group membership on alcohol reduction (see Table 3).

**Table 3:** Comparisons across alcohol intervention, tailored list only; alcohol intervention, dynamic referral; and control conditions.

Characteristic			Intervention-provider list (n=83), n (%)	Intervention-dynamic referral (n=14), n (%)	Control (n=115), n (%)
**Outpatient alcohol abuse treatment**					
	Contact with alcohol abuse treatment provider				
		Contact at 1 month	3 (4)	4 (29)	10 (8.7)
		Contact at 3 months	5 (6)	8 (57)	13 (11.3)
	Initiated treatment (evaluated by alcohol abuse treatment provider)				
		Treatment initiation at 1 month	1 (1)	2 (14)	7 (6.1)
		Treatment initiation at 3 months	2 (2)	4 (29)	8 (7.0)
	Treatment engagement, either time		1 (1)	2 (14)	8 (7.0)
	Treatment completion		1 (1)	2 (14)	7 (6.1)
**Alcohol use behavior**					
	Used alcohol (since ED^a^ visit)				
		Abstinent for first month (since visit)	8 (10)	0 (0)	12 (10.4)
		Abstinent for 3 months (since visit)	3 (4)	0 (0)	4 (3.5)
		At least one quit attempt at 1 month	15 (18)	2 (14)	37 (32.2)
		At least one quit attempt at 3 months	27 (33)	3 (21)	58 (50.4)
		Attempted to reduce use at 1 month	21 (25)	4 (29)	45 (39.1)
		Attempted to reduce use at 3 months	29 (35)	4 (29)	57 (49.6)

^a^ED: emergency department.

## Discussion

### Principal Findings

ED-originated alcohol interventions have potential for substantial public health impact by offering widespread SBIRT for risky alcohol use within a population that is both high risk and difficult to reach [4,10,13]. However, many challenges continue to impede the adoption of interventions into routine clinical care, including competing time demands and priorities, a focus on acute care, and insufficient specialized training of providers in risky alcohol use interventions. Technology facilitated intervention models that maximize efficiency and relieve clinician burden may offer a solution.

The results of this clinical trial exploring the benefits of using a single administration, stand-alone computerized intervention were mixed. All participants scored positive for risky alcohol use, and therefore received a patient feedback report with personalized information and referrals. Those who reported not currently being in treatment and who reported some desire to change their drinking were offered a dynamic referral. Although overall no significant differences were observed between conditions for contact with a treatment provider, treatment initiation, treatment engagement, and treatment completion, a closer look at the data suggests that the dynamic referral may still hold promise for promoting treatment engagement. Subanalyses revealed that among the experimental participants, those who accepted a dynamic referral were more likely to make contact with a treatment provider and have higher rates of treatment initiation than control participants. However, these effects did not lead to continued engagement in treatment or changes in alcohol use over the 3-month period following the ED visit. Moreover, some of the trends for attempting to change, such as reporting any attempt to reduce use, favored the control condition, rather than the intervention condition, though these differences were not statistically different. Additional research is needed to probe this pattern to establish if there may be an iatrogenic impact of providing personalized information and referrals in dampening self-change.

There are several factors that may have hampered the HERA’s impact on treatment and alcohol use behavior. One factor may be a lack of adoption and implementation by ED clinical staff. Although the clinical staff members who received the health care provider reports were trained to interpret the findings, they were not specifically trained or mandated to provide counseling or additional intervention materials to patients as a result of reviewing the report. Analyses indicated that clinical staff did not provide additional counseling or intervention materials to participants in the intervention group, which could be interpreted as weak clinician adoption or support of the intervention. Although the HERA is designed to offer brief intervention and referral to treatment as a stand-alone service, a cooperative approach which includes protocols for clinician involvement in response to a positive screen on the health care provider report may prove a stronger intervention than a stand-alone automated referral. Furthermore, the sample was heterogeneous, with only a minority scoring in the severe range on the AUDIT (low to moderate risk, n=173/212 [82%], moderate to high risk, n=13/212 [6.1%], and high to very high risk, n=26/212 [12.3%]). This undoubtedly dampens the level of interest in specialized treatment.

An additional factor limiting clinical impact could be the low-intensity nature of the HERA intervention. The HERA was designed as a one-time, brief interaction due to the fast-paced ED environment filled with competing demands for time and resources. Minimizing the intervention for this purpose could have adversely affected the HERA’s potential for clinical impact. The brief encounter with the HERA, while efficient and time-saving for clinicians, may not be powerful enough to support long-term changes in alcohol use behavior. Future technology-facilitated interventions may need to integrate motivational tools for behavior change, such as Web-based multimedia content or longitudinal interaction beyond the ED visit.

A final factor that may have impeded continued treatment and change in risky alcohol use behavior are barriers related to patient follow-through, including a lack of transportation or childcare, fees for services, and schedule conflicts. Although the dynamic referral was designed to connect patients with a “best match” treatment facility based on personal characteristics, the scope is limited to general characteristics such as location, insurance provider, and desire for telephone or in-person treatment. Motivated patients, who initiated contact with a nearby treatment provider compatible with their insurance carrier, may still have been unable to attend treatment due to the aforementioned circumstances [41]. Additionally, individuals were offered free alternatives to fee-for-service treatment models to help address cost barriers, but free treatment providers may not have been conveniently located or available during hours conducive to every patient’s schedule.

### Limitations

Several limitations exist that impact interpreting the results. First, because a minimal treatment control group was used, rather than true treatment as usual, the assessment and resource list provided to the minimal treatment control group may have had an intervention effect and artificially inflated treatment contact and behavior change in the control group. Second, the use of a modified AUDIT allowed for time-sensitive brief assessment of alcohol use, but assessed use over a shorter period than other methods, such as the Timeline Follow Back [42], and may not have allowed for a large enough assessment window to detect risky alcohol use in some individuals. Third, results may have been skewed by the nature of self-report due to the ambiguity inherent in measuring alcohol consumption by number of drinks. Although standard measurements have been outlined by the AUDIT [35], and clear examples of “one alcoholic drink” were provided during the assessment, participant understanding of the size and volume of “one alcoholic drink” varies considerably. Finally, by focusing solely on alcohol users, who used alcohol above the AUDIT quantity or frequency guidelines and who had not used illicit drug in the past 12 months, the generalizability of the results is limited. Future research should examine the efficacy of automated referral systems for alcohol treatment among all alcohol users.

The fact that very few participants accepted the dynamic referral highlights a potential limitation of the HERA model itself. Although participants who accepted a dynamic referral were more likely to contact a treatment provider and demonstrated higher rates of treatment initiation than control participants, impact will be minimal unless more patients begin accepting the referral. Future studies of similar models should aim to identify and overcome barriers to referral acceptance. A final limitation is that participants who failed to follow-through with treatment after receiving the referral were not questioned as to what factors contributed to their failure to follow-through. Costs associated with fee-for-service treatment options may have been a barrier to treatment initiation and engagement, although potentially alleviated by the inclusion of free treatment options in addition to the fee-for-service selections. Barriers to patient follow-through in systems like the HERA should be explored in future studies.

### Conclusions

The HERA aims to satisfy clinical practice mandates for SBIRT for risky alcohol users in the ED setting. For those who accepted the dynamic referral, the HERA was effective at promoting contact with an alcohol treatment provider and initiating risky alcohol use treatment. Unfortunately, when employed as a stand-alone intervention, the HERA did not lead to sustained treatment engagement or changes in alcohol use during the 3 months following the initial ED visit. These results raise two questions: (1) Do stand-alone, brief, automated interventions lack the power to sufficiently motivate sustained alcohol use treatment engagement and behavior change? and (2) Is SBIRT for risky alcohol use satisfactory for all populations, particularly those unable or unwilling to pay fees associated with treatment services or underserved populations with limited access to health care, as represented in this study? This study highlights the need for developing and studying interventions that work alongside alcohol treatment linkage strategies. The prototype of the HERA was called the Dynamic Assessment and Referral System for Substance Abuse (DARSSA). The name was changed to reflect our long-term plans to expand the system to provide SBIRT for other nonsubstance problems, like depression and interpersonal violence.

## Appendix

Multimedia Appendix 1 Sample assessment screenshots.

Multimedia Appendix 2 Sample patient feedback report.

Multimedia Appendix 3 Total Health evaluation and referral assistant (HERA) potential participants.

Multimedia Appendix 4 Demographic characteristics of the analyzed sample.

Multimedia Appendix 5 CONSORT eHealth checklist.

Multimedia Appendix 6 CONSORT-EHEALTH checklist V1.6.2.

## Abbreviations

AUDIT: Alcohol Use Disorders Identification Test
ED: emergency department
GEE: generalized estimating equation
HERA: Health Evaluation and Referral Assistant
OR: odds ratio
PHQ-2: Patient Health Questionaire-2
RA: research assistant
RCT: randomized controlled trial
SBI: screening and brief intervention
SBIRT: screening, brief intervention, and referral to treatment
SD: standard deviation

## References

[1] Gonzales K, Roeber J, Kanny D, Tran A, Saiki C, Johnson H, Yeoman K, Safranek T, Creppage K, Lepp A, Miller T, Tarkhashvili N, Lynch KE, Watson JR, Henderson D, Christenson M, Geiger SD, Centers for Disease Control and Prevention (CDC) (2014). Alcohol-attributable deaths and years of potential life lost--11 States, 2006-2010. MMWR Morb Mortal Wkly Rep.

[2] Sanjuan PM, Rice SL, Witkiewitz K, Mandler RN, Crandall C, Bogenschutz MP (2014). Alcohol, tobacco, and drug use among emergency department patients. Drug Alcohol Depend.

[3] Centers for Disease Control and Prevention (CDC) (2012). Vital signs: binge drinking prevalence, frequency, and intensity among adults - United States, 2010. MMWR Morb Mortal Wkly Rep.

[4] Cherpitel CJ, Ye Y (2008). Drug use and problem drinking associated with primary care and emergency room utilization in the US general population: data from the 2005 national alcohol survey. Drug Alcohol Depend.

[5] Cunningham RM, Bernstein SL, Walton M, Broderick K, Vaca FE, Woolard R, Bernstein E, Blow F, D'Onofrio G (2009). Alcohol, tobacco, and other drugs: future directions for screening and intervention in the emergency department. Acad Emerg Med.

[6] Centers for Disease Control and Prevention (CDC) CDC.

[7] Hankin A, Daugherty M, Bethea A, Haley L (2013). The Emergency Department as a prevention site: a demographic analysis of substance use among ED patients. Drug Alcohol Depend.

[8] Rockett IR, Putnam SL, Jia H, Chang CF, Smith GS (2005). Unmet substance abuse treatment need, health services utilization, and cost: a population-based emergency department study. Ann Emerg Med.

[9] Rockett IR, Putnam SL, Jia H, Smith GS (2003). Assessing substance abuse treatment need: a statewide hospital emergency department study. Ann Emerg Med.

[10] Lowenstein SR, Koziol-McLain J, Thompson M, Bernstein E, Greenberg K, Gerson LW, Buczynsky P, Blanda M (1998). Behavioral risk factors in emergency department patients: a multisite survey. Acad Emerg Med.

[11] Keyes KM, Liu XC, Cerda M (2012). The role of race/ethnicity in alcohol-attributable injury in the United States. Epidemiol Rev.

[12] Williams EC, Gupta S, Rubinsky AD, Jones-Webb R, Bensley KM, Young JP, Hagedorn H, Gifford E, Harris AH (2016). Racial/ethnic differences in the prevalence of clinically recognized alcohol use disorders among patients from the U.S. Veterans Health Administration. Alcohol Clin Exp Res.

[13] Murphy MK, Bijur PE, Rosenbloom D, Bernstein SL, Gallagher EJ (2013). Feasibility of a computer-assisted alcohol SBIRT program in an urban emergency department: patient and research staff perspectives. Addict Sci Clin Pract.

[14] Substance AbuseMental Health Services Administration, Department of HealthHuman Services (2013). SAMHSA.

[15] Field CA, Baird J, Saitz R, Caetano R, Monti PM (2010). The mixed evidence for brief intervention in emergency departments, trauma care centers, and inpatient hospital settings: what should we do?. Alcohol Clin Exp Res.

[16] Babor TF, McRee BG, Kassebaum PA, Grimaldi PL, Ahmed K, Bray J (2007). Screening, brief intervention, and referral to treatment (SBIRT): toward a public health approach to the management of substance abuse. Subst Abus.

[17] Zarkin GA, Bray JW, Davis KL, Babor TF, Higgins-Biddle JC (2003). The costs of screening and brief intervention for risky alcohol use. J Stud Alcohol.

[18] Academic ED SBIRT Research Collaborative (2007). The impact of screening, brief intervention, and referral for treatment on emergency department patients' alcohol use. Ann Emerg Med.

[19] U.S. Preventive Services Task Force (2004). Screening and behavioral counseling interventions in primary care to reduce alcohol misuse: recommendation statement. Ann Intern Med.

[20] Substance Abuse and Mental Health Services Administration, Department of Health and Human Services (2006). SAMHSA.

[21] Substance Abuse and Mental Health Services Administration, Department of Health and Human Services (2012). SAMHSA.

[22] McKnight-Eily LR, Liu Y, Brewer RD, Kanny D, Lu H, Denny CH, Balluz L, Collins J, Centers for Disease Control and Prevention (CDC) (2014). Vital signs: communication between health professionals and their patients about alcohol use--44 states and the District of Columbia, 2011. MMWR Morb Mortal Wkly Rep.

[23] Boudreaux ED, Abar B, Haskins B, Bauman B, Grissom G (2015). Health evaluation and referral assistant: a randomized controlled trial to improve smoking cessation among emergency department patients. Addict Sci Clin Pract.

[24] Cunningham RM, Harrison SR, McKay MP, Mello MJ, Sochor M, Shandro JR, Walton MA, D'Onofrio G (2010). National survey of emergency department alcohol screening and intervention practices. Ann Emerg Med.

[25] Terrell F, Zatzick DF, Jurkovich GJ, Rivara FP, Donovan DM, Dunn CW, Schermer C, Meredith JW, Gentilello LM (2008). Nationwide survey of alcohol screening and brief intervention practices at US Level I trauma centers. J Am Coll Surg.

[26] Boudreaux ED, Abar B, Baumann BM, Grissom G (2013). A randomized clinical trial of the health evaluation and referral assistant (HERA): research methods. Contemp Clin Trials.

[27] Etter JF, Perneger TV (2001). Effectiveness of a computer-tailored smoking cessation program: a randomized trial. Arch Intern Med.

[28] Holtz K, Landis R, Nemes S, Hoffman J (2001). Development of a computerized screening system to identify substance abuse in primary care. J Healthc Qual.

[29] Prochaska JO, Velicer WF, Fava JL, Ruggiero L, Laforge RG, Rossi JS, Johnson SS, Lee PA (2001). Counselor and stimulus control enhancements of a stage-matched expert system intervention for smokers in a managed care setting. Prev Med.

[30] Revere D, Dunbar PJ (2001). Review of computer-generated outpatient health behavior interventions: clinical encounters “in absentia”. J Am Med Inform Assoc.

[31] Rhodes KV, Lauderdale DS, Stocking CB, Howes DS, Roizen MF, Levinson W (2001). Better health while you wait: a controlled trial of a computer-based intervention for screening and health promotion in the emergency department. Ann Emerg Med.

[32] Velicer WF, Prochaska JO, Fava JL, Laforge RG, Rossi JS (1999). Interactive versus noninteractive interventions and dose-response relationships for stage-matched smoking cessation programs in a managed care setting. Health Psychol.

[33] Zeiler CA, Nemes S, Holtz KD, Landis RD, Hoffman J (2002). Responses to a drug and alcohol problem assessment for primary care by ethnicity. Am J Drug Alcohol Abuse.

[34] Boudreaux ED, Bedek KL, Gilles D, Baumann BM, Hollenberg S, Lord SA, Grissom G (2009). The Dynamic Assessment and Referral System for Substance Abuse (DARSSA): development, functionality, and end-user satisfaction. Drug Alcohol Depend.

[35] Saunders JB, Aasland OG, Babor TF, de la Fuente JR, Grant M (1993). Development of the alcohol use disorders identification test (AUDIT): WHO collaborative project on early detection of persons with harmful alcohol consumption-II. Addiction.

[36] Babor TE, Higgins-Biddle J, Dauser D, Higgins P, Burleson JA (2005). Alcohol screening and brief intervention in primary care settings: implementation models and predictors. J Stud Alcohol.

[37] (2014). mail.umassmed.edu.

[38] Kroenke K, Spitzer RL, Williams JB (2003). The Patient Health Questionnaire-2: validity of a two-item depression screener. Med Care.

[39] William MR, Rollnick S (2013). Motivational Interviewing, Third Edition: Helping People Change (Applications of Motivational Interviewing).

[40] Prochaska JO, DiClemente CC, Norcross JC (1992). In search of how people change: applications to addictive behaviors. Am Psychol.

[41] Boudreaux ED, Baumann BM, Perry J, Marks D, Francies S, Camargo CA, Ziedonis D (2008). Emergency department initiated treatments for tobacco (EDITT): a pilot study. Ann Behav Med.

[42] Sobell L, Sobell M National Institutes of Health, National Institute on Alcohol Abuse and Alcoholism.

