# Supplementary material for: Health Evaluation and Referral Assistant: A Randomized Controlled Trial of a Web-Based Screening, Brief Intervention, and Referral to Treatment System to Reduce Risky Alcohol Use Among Emergency Department Patients
Source: J Med Internet Res. 2017 May 1;19(5):e119. doi: 10.2196/jmir.6812 (PMC5432666; doi:10.2196/jmir.6812)
Supplement: Multimedia Appendix 1 [file jmir_v19i5e119_app1.pdf]

UMassMemorial Health Care

powered by polaris HEALTH DIRECTIONS

em Português | en Español

PROGRESS

23%

PAUSE SURVEY

How often do you have a drink containing alcohol?

- Never
- Less than monthly
- Monthly
- Weekly (once a week)
- 2 - 3 times a week
- 4 - 6 times a week
- Daily
- I prefer not to answer

BACK NEXT

UMassMemorial Health Care

powered by polaris HEALTH DIRECTIONS

em Português | en Español

PROGRESS

24%

PAUSE SURVEY

A standard drink is one 12-ounce can or bottle of beer, one glass of wine, one cocktail / mixed drink, or one shot of liquor.
How many standard drinks containing alcohol do you have on a typical day when you are drinking?

- 1 drink
- 2 drinks
- 3 drinks
- 4 drinks
- 5-6 drinks
- 7-9 drinks
- 10 or more drinks
- I prefer not to answer

BACK NEXT

UMassMemorial Health Care

powered by polaris HEALTH DIRECTIONS

em Português | en Español

PROGRESS

25%

PAUSE SURVEY

How often do you have four or more drinks on one occasion?

- Never
- Less than monthly
- Monthly
- Weekly (once a week)
- 2 - 3 times a week
- 4 - 6 times a week
- Daily
- I prefer not to answer

BACK NEXT

UMassMemorial Health Care

powered by polaris HEALTH DIRECTIONS

em Português | en Español

PROGRESS

60%

PAUSE SURVEY

How often during the last year have you found that you were not able to stop drinking once you had started?

- Never
- Less than monthly
- Monthly
- Weekly
- Daily or almost daily

BACK NEXT

UMassMemorial Health Care

powered by polaris HEALTH DIRECTIONS

em Português | en Español

PROGRESS

61%

PAUSE SURVEY

How often during the last year have you failed to do what was normally expected of you because of drinking?

- Never
- Less than monthly
- Monthly
- Weekly
- Daily or almost daily

BACK NEXT

UMassMemorial Health Care

powered by polaris HEALTH DIRECTIONS

em Português | en Español

PROGRESS

61%

PAUSE SURVEY

How often during the last year have you needed a first drink in the morning to get yourself going after a heavy drinking session?

- Never
- Less than monthly
- Monthly
- Weekly
- Daily or almost daily

BACK NEXT

UMassMemorial Health Care

powered by polaris HEALTH DIRECTIONS

em Português | en Español

PROGRESS

62%

PAUSE SURVEY

How often during the last year have you had a feeling of guilt or remorse after drinking?

- Never
- Less than monthly
- Monthly
- Weekly
- Daily or almost daily

BACK NEXT

UMassMemorial Health Care

powered by polaris HEALTH DIRECTIONS

em Português | en Español

PROGRESS

63%

PAUSE SURVEY

How often during the last year have you been unable to remember what happened the night before because of your drinking?

- Never
- Less than monthly
- Monthly
- Weekly
- Daily or almost daily

BACK NEXT

UMassMemorial Health Care

powered by polaris HEALTH DIRECTIONS

em Português | en Español

PROGRESS

64%

PAUSE SURVEY

Have you or someone else been injured because of your drinking?

- No
- Yes, but not in the last year
- Yes, during the last year

BACK NEXT

UMassMemorial Health Care

powered by polaris HEALTH DIRECTIONS

em Português | en Español

PROGRESS

65%

PAUSE SURVEY

Has a relative, friend, doctor or other health care worker been concerned about your drinking or suggested you cut down?

- No
- Yes, but not in the last year
- Yes, during the last year

BACK NEXT

UMassMemorial Health Care

powered by polaris HEALTH DIRECTIONS

em Português | en Español

PROGRESS

66%

PAUSE SURVEY

How long has it been since you last drank any alcohol?

- Today
- 1-2 days
- 3-7 days
- 8-30 days
- More than 30 days

BACK NEXT

UMassMemorial Health Care

powered by polaris HEALTH DIRECTIONS

em Português | en Español

PROGRESS

67%

PAUSE SURVEY

Have you ever been in treatment for alcohol use?

- No
- Yes, but I AM NOT CURRENTLY in treatment
- Yes, and I AM CURRENTLY in treatment

BACK NEXT
