# Supplementary material for: Health Evaluation and Referral Assistant: A Randomized Controlled Trial of a Web-Based Screening, Brief Intervention, and Referral to Treatment System to Reduce Risky Alcohol Use Among Emergency Department Patients
Source: J Med Internet Res. 2017 May 1;19(5):e119. doi: 10.2196/jmir.6812 (PMC5432666; doi:10.2196/jmir.6812)
Supplement: Multimedia Appendix 2 [file jmir_v19i5e119_app2.pdf]

PLEASE GIVE THIS TO THE PATIENT

Patient: BROWN, MS
DOB: 07/12/1990 (26 yo)
Date: 01/10/2017
Location: RBIRT test site

Your health and your behavior

You answered some questions about your health and health behaviors.

This summary is a personal breakdown of your health risk. Also, it provides some useful information on how you can get help, if you want it. A shorter report has been printed for the healthcare team that put you in touch with the telephone counselor. We encourage you to talk with your healthcare providers about any concerns you have. They can help you learn more about how your health behaviors may be affecting your health.

You stated that you currently or recently have used alcohol.

Some people do not think their use is a problem, while others do. Where do you fall? Is your use working for you or against you? Many people find that quitting improves their lives. They are healthier. They have more energy, more money, and better relationships. What are the most important goals in your life? Would changing your behaviors help you to get those things most important to you?

YOUR RESULTS:

You've answered some questions about your health, behaviors, and substance use. Here are your personalized results:

DRUGS: **Not determined**

ALCOHOL: **Moderate to High risk**

TOBACCO: **Not determined**

Where can I get help if I want it?

You said you are not interested in changing your alcohol use. A list of treatment providers appears below, in case you decide later that you want help changing your alcohol use.

Alcohol Treatment Providers

1. **AdCare Hospital**
   (508) 453-3053
   95 Lincoln Street,
   Worcester, MA (outpatient)
2. **Spectrum Health Systems Inc**
   (508) 854-3320x1161
   585 Lincoln Street,
   Worcester, MA (outpatient)
3. **Spectrum Health Systems Inc**
   (508) 797-6100
   105 Merrick Street,
   Worcester, MA (outpatient)

Other Resources

1. **Alcoholics Anonymous**
   1-212-870-3400
   http://www.aa.org
2. **Substance Abuse and Mental Health Services Administration**
   1-800-662-4357
   http://dasis3.samhsa.gov

Your alcohol use in more detail

You answered some questions about how much you drink and about problems that are sometimes related to drinking. The scale below shows your alcohol abuse risk level:

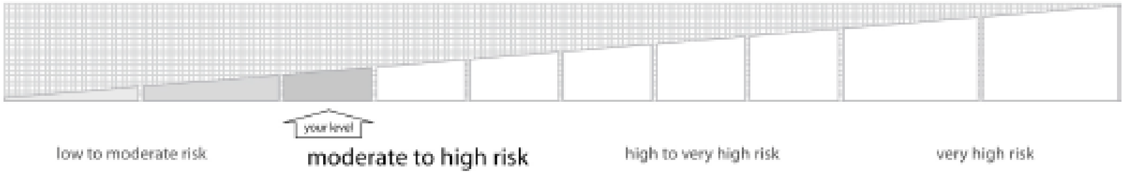

Based on your answers, you are at moderate to high risk for developing alcohol-related problems.

This level of risk has been determined by several large research studies. Your scores were compared to people who had drinking problems or who later developed them. You scored the same as people who drink more than the recommended amount or who already have some negative effects of use. It is common for people who score in this range to have increased negative effects if they don't quit or cut back on their drinking. The best way to avoid problems related to alcohol use is to watch how much and how often you drink. In addition, the speed that you drink is important. An average person who drinks more than 1 drink an hour is likely to have a blood alcohol level above the "drunk driving" cut-off in most states. Even if you do not think you are drunk, you could still be arrested for drunk driving if stopped.

If you are pregnant, you should seriously consider not drinking at all. There is no known "safe level" of drinking for pregnant women.

Based on years of research, the National Institute for Alcohol Abuse and Alcoholism has found that women who drink more than 7 drinks a week, or who have more than 3 drinks on a given occasion, are at risk for developing drinking problems.

Is My Drinking Harmful?

Many people who drink alcohol wonder about whether their use is harming them in some way. It is important to remember three facts when you are thinking about whether your drinking is hurting you or someone you love:

1. Regular use of alcohol above recommended amounts can have both short-term and long-term negative effects
2. The harmful effects may not always be noticeable at first, but they can still be there
3. Some effects can be lasting, even if you only drink to excess every once in a while. For example, even occasional drinking and driving can put you at risk for a crash

Alcohol:

Heavy drinking can increase your risk for many illnesses and other problems, including:

- Certain cancers, like stomach cancer
- Stomach and other gastro-intestinal problems
- Liver disease
- Immune system problems (makes you more likely to get sick)
- Problems with memory and concentration
- Increased risks for accidents and falls
- Depression
- Troubled relationships
- Birth defects (in pregnant women)

The Scales - which way are you tipping?

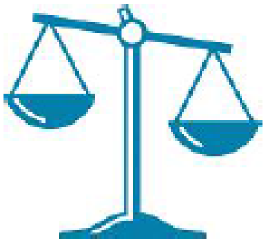

You said you were not interested in changing your drinking at this time. You may change your mind someday, but either way, it has to be your decision. Most people who are not ready to change do not believe that the benefits of change outweigh the benefits of continuing to use. Sometimes they really haven't thought about it much. Even people who are not ready to change now sometimes find it useful to complete the "scales" exercise.

1. In the left column below, you will find the good things you described about using alcohol (what you like about it).

2. In the right column, are the not so good things you listed about usingalcohol.

3. Are the problems caused by alcohol outweighing the benefits?

4. Are the good things about using alcohol more important to you than the not so good things about using alcohol? What course of action is most in line with your most important values?

Good things about using alcohol	Good things about quitting alcohol
Feels good (fun, high, happy)	Health problems (illness, withdrawals, hangovers)
Relax (helps cope, stress relief	Social problems (fights hurts/disappoints others)
Social (friends, family, parties, holidays)	

Breaking the chains.

One thing that makes changing difficult is that using alcohol gets "chained" to certain people, places, things, emotions, and situations. These become triggers to use and can lead to intense cravings, making you more likely to lapse. To help identify and cope with these triggers, complete the steps below.

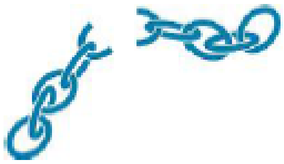

1. List all of the people, places, things, emotions, and situations that trigger you to use alcohol.
2. In the left column below, write the 3 that are most difficult for you to resist.
3. In the right column, write your plan to avoid or manage these triggers besides using.

1. People, places, things, emotions and situations that trigger you to use alcohol

2. Most difficult to resist		3. Plan to avoid or manage triggers
1.	→	1.
2.	→	2.
3.	→	3.

Stairway to Change - where are you?

You said that you are not interested in cutting back or quitting your alcohol use. That decision is up to you. If you change your mind, you may find the "Stairway" helpful.
Most people are more successful if they create a plan. If you decide to cut back or quit and find you are having trouble achieving your goals, you should consider getting into treatment. The good news is that there are many very effective therapies to change your alcohol use. See Page 1 for a list of providers near you.

1. Start from the bottom. Check off each step that you can agree to do.

2. Post this sheet in a place you will see it often, like a refrigerator.

3. Now do them! Review the stairway to change and exercises below every day.

- 8 ☐ Maintain abstinence and stay off alcohol forever!
- 7 ☐ Quit drinking! My quit date will be: ________________.
- 6 ☐ Cut back on my drinking
- 5 ☐ Begin treatment or a self-help program.
- 4 ☐ Find out more about treatment options and other sources of help (see Page 1)
- 3 ☐ Come up with a plan to better handle my "triggers" (see Breaking the Chains exercise).
- 2 ☐ Talk to doctor
- 1 ☑ Seriously review my reasons for changing my alcohol use (see Scales exercise).
