# Supplementary figures and images for: Health Evaluation and Referral Assistant: A Randomized Controlled Trial of a Web-Based Screening, Brief Intervention, and Referral to Treatment System to Reduce Risky Alcohol Use Among Emergency Department Patients
Source: J Med Internet Res. 2017 May 1;19(5):e119. doi: 10.2196/jmir.6812 (PMC5432666; doi:10.2196/jmir.6812)

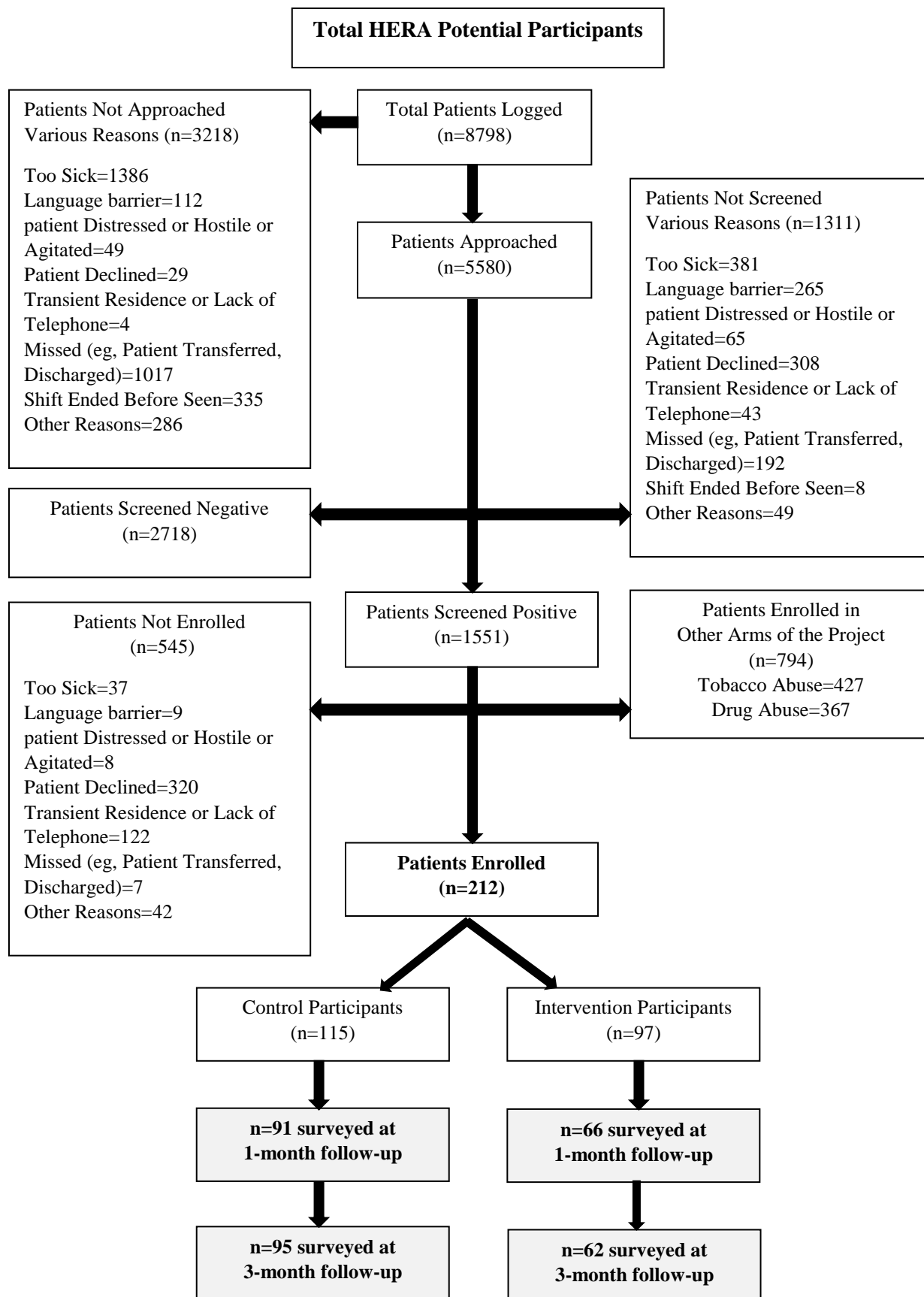

Supplement: Multimedia Appendix 3 [file jmir_v19i5e119_app3.pdf]
