# Supplementary material for: Health Evaluation and Referral Assistant: A Randomized Controlled Trial of a Web-Based Screening, Brief Intervention, and Referral to Treatment System to Reduce Risky Alcohol Use Among Emergency Department Patients
Source: J Med Internet Res. 2017 May 1;19(5):e119. doi: 10.2196/jmir.6812 (PMC5432666; doi:10.2196/jmir.6812)
Supplement: Multimedia Appendix 4 [file jmir_v19i5e119_app4.pdf]

Demographic characteristics[a]		Frequency (%)		Mean (SD[b])	
**Baseline AUDIT[c] sum score**				10.96 (6.59)	
**AUDIT risk severity**					
	Low to moderate risk	173 (82%)			
	Moderate to high risk	13 (6%)			
	High to very high risk	26 (12%)			
		Experimental (n=97)		Control (n=115)	
		Frequency (%)	Mean (SD)	Frequency (%)	Mean (SD)
**Participant sex**					
	Male	67 (69%)		67 (58%)	
	Female	30 (31%)		48 (42%)	
**Participant age**			37.3 (12.5)		38.7 (14.1)
**Data collection site**					
	Cooper University Hospital (NJ[d])	26 (27%)		35 (30%)	
	UMass University Hospital (MA[e])	70 (72%)		70 (61%)	
	UMass Memorial Hospital (MA)	1 (1%)		7 (6%)	
	Marlborough Hospital (MA)	0 (0%)		3 (3%)	
**Race**					
	White	77 (79%)		93 (81%)	
	Black	15 (16%)		18 (16%)	
	Other or undocumented	6 (6%)		3 (2%)	
**Ethnicity**					
	Non-Hispanic or Latino	79 (44%)		99 (86%)	
	Hispanic or Latino	14 (14%)		12 (10%)	
	Not documented	4 (4%)		4 (3%)	

**Insurance status**[f]					
	Private insurance	34 (35%)		39 (34%)	
	Medicaid	6 (6%)		21 (18%)	
	State financed—non-Medicaid	21 (22%)		18 (16%)	
	Medicare	12 (12%)		16 (14%)	
	Other kind of insurance not listed	10 (10%)		12 (10%)	
	No insurance	16 (17%)		18 (16%)	
**Readiness to change**					
	No	52 (54%)		56 (49%)	
	Undecided	24 (25%)		17 (15%)	
	Yes, I would like to cut back	10 (10%)		23 (20%)	
	Yes, I would like to quit completely	11 (11%)		19 (17%)	
**Treatment history**					
	No	82 (85%)		99 (86%)	
	Yes, but I am not currently in treatment	12 (12%)		11 (10%)	
	Yes, but I am currently in treatment	3 (3%)		5 (4%)	
**Readiness to enter treatment**					
	No	10 (10%)		0 (0%)	
	Yes	8 (8%)		0 (0%)	
**Withdrawal symptoms**					
	Seizures or convulsions	0 (0%)		1 (1%)	
	Hallucinations	0 (0%)		1 (1%)	
	Confusion or disorientation	3 (3%)		2 (2%)	
	Paranoid thinking	3 (3%)		3 (3%)	
	Severe depression	3 (3%)		3 (3%)	
	Severe loss of energy (lethargy)	3 (3%)		9 (8%)	
	None of the above	1 (1%)		4 (4%)	
**Frequency of alcohol use**					
	Less than monthly	2 (2%)		2 (2%)	

	Monthly	15 (16%)		17 (15%)	
	Weekly	35 (36%)		28 (24%)	
	2-3 times a week	25 (26%)		29 (25%)	
	4-6 times a week	5 (5%)		15 (13%)	
	Daily	15 (16%)		24 (21%)	
**Number of drinks on typical drinking days**			5.74 (3.36)		5.74 (4.81)
**Frequency of four or more drinks on one occasion**					
	Never	2 (2%)		2 (2%)	
	Less than monthly	8 (8%)		13 (11%)	
	Monthly	33 (34%)		32 (28%)	
	Weekly	32 (33%)		32 (28%)	
	2-3 times a week	13 (13%)		25 (22%)	
	4-6 times a week	3 (3%)		4 (4%)	
	Daily	6 (6%)		7 (6%)	
**Referral**[g]					
	Tailored, printed list of providers (only)	83 (86%)			
	Dynamic referral accepted	14 (14%)			

[a]Illicit drug users were excluded from the alcohol sample; they were enrolled and randomized into a separate sample, to be reported at a later date.

[b]SD: standard deviation.

[c]AUDIT: Alcohol Use Disorders Identification Test.

[d]NJ: New Jersey.

[e]MA: Massachusetts.

[f]Insurance status categories are not mutually exclusive (ie, participants can have multiple types of insurance).

[g]Only participants in the intervention condition. All patients in the intervention group received the tailored, printed list of providers by default. Dynamic referral consists of a faxed referral with a brief alcohol use summary to an alcohol abuse treatment provider matched to the individual based on location of residence and preference for telephone versus in-person treatment. All patients in the intervention group received the tailored, printed list of providers by default.
